# High-density HNIW/TNT cocrystal synthesized using a green chemical method

**DOI:** 10.1107/S2052520618008442

**Published:** 2018-07-23

**Authors:** Yan Liu, Chongwei An, Jin Luo, Jingyu Wang

**Affiliations:** a School of Environment and Safety Engineering, North University of China, Taiyuan, Shanxi 030051, People’s Republic of China

**Keywords:** HNIW, TNT, HNIW/TNT cocrystal, high density, green chemical method, energetic materials

## Abstract

The main challenge for achieving better energetic materials is to increase their density. In this paper, cocrystals of HNIW (2,4,6,8,10,12-hexa­nitro-2,4,6,8,10,12-hexa­aza­isowurtzitane, often referred to as CL-20) with TNT (2,4,6-tri­nitro­toluene) were synthesized using ethanol in a green chemical method. The cocrystal was formulated as C_13_H_11_N_15_O_18_ and possesses a higher density (1.934 g cm^−3^) than published previously (1.846 g cm^−3^). This high-density cocrystal possesses a new structure, which can be substantiated by the different types of hydrogen bonds. The predominant driving forces that connect HNIW with TNT in the new cocrystal were studied at ambient conditions using single-crystal X-ray diffraction, powder X-ray diffraction, Fourier transform–infrared spectroscopy and Raman spectroscopy. The results reveal that the structure of the new HNIW/TNT cocrystals consists of three one-dimensional hydrogen-bonded chains exploiting the familiar HNIW–TNT multi-component supramolecular structure, in which two hydrogen-bonded chains are between —NO_2_ (HNIW) and —CH (TNT), and one hydrogen-bonded chain is between —CH (HNIW) and —NO_2_ (TNT). The changes to the electron binding energy and type of element in the new cocrystal were traced using X-ray photoelectron spectroscopy. Meanwhile, the physicochemical characteristics alter after cocrystallization due to the hydrogen bonding. It was found that the new HNIW/TNT cocrystal is more thermodynamically stable than HNIW. Thermodynamic aspects of new cocrystal decomposition are investigated in order to explain this observation. The detonation velocity of new HNIW/TNT cocrystals is 8631 m s^−1^, close to that of HNIW, whereas the mechanical sensitivity is lower than HNIW.

## Abstract 2

HNIW/TNT cocrystals are synthesized by a new chemical method. The performance of the cocrystals is improved from previous preparation techniques and this new method is a more environmentally friendly approach.

## 1. Introduction   

Currently, development of new energetic materials with insensitive high explosives is of great interest for applications in civil constructions, explosives, rocket propellants and airbag inflators (Liu *et al.*, 2015 ▸). For this reason, cocrystallization has attracted considerable attention due to its ability to modify and optimize the physicochemical properties of energetic materials (Bennion *et al.*, 2015 ▸; Wu *et al.*, 2015 ▸). A cocrystal can be defined as multicomponent crystals of neutral molecular species in a specified ratio and is generally stabilized by hydrogen bonding, π–π stacking, *p*–π stacking, halogen bonds and van der Waals forces, instead of the common chemical bond (Bond, 2007 ▸; Stahly, 2009 ▸; Landenberger & Matzger, 2012 ▸). This new crystal engineering has been successfully applied broadly in the fields of explosives, propellants and pyrotechnics, and is recognized as a powerful tool for altering properties of existing energetic materials to create less-sensitive explosives (Shi *et al.*, 2016 ▸; Sun *et al.*, 2018 ▸).

HNIW, a non-aromatic cyclic nitramine, is a typical high-energy-density explosive. Higher specific impulse, ballistics, oxygen balance, detonation velocity and detonation pressure make it a better energetic material than other nitramines. However, HNIW displays sensitivity to high impact, friction, shock waves and electric sparks which makes it seriously limited for civilian application and military use (Sun *et al.*, 2018 ▸; Turcotte *et al.*, 2005 ▸; Liu *et al.*, 2016 ▸). TNT possesses low density, oxygen balance, detonation velocity and detonation pressure and is defined as an insensitive energetic material (Wilson *et al.*, 1990 ▸). Therefore, HNIW/TNT cocrystals have been synthesized *via* a cocrystal strategy. HNIW/TNT cocrystals improve the sensitivity of the HNIW but the energy output is better still. The chemical structures of the cocrystal coformers, HNIW and TNT, are shown in Scheme (1).[Chem scheme1] 
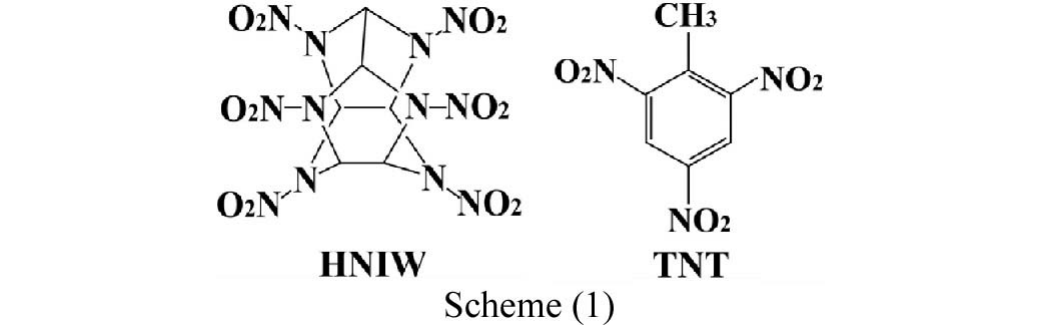


In recent years, solution crystallization methods have been used to obtain cocrystals (Xu *et al.*, 2015 ▸; Bennion *et al.*, 2017 ▸; Yang *et al.*, 2013 ▸). A suitable organic solvent was used to directly dissolve the whole cocrystal coformers, but these organic solvents are commonly toxic (Lin *et al.*, 2016 ▸; Gao *et al.*, 2017 ▸; Ghosh *et al.*, 2012 ▸). An obvious disadvantage of this method is that the application of large amounts of toxic organic solvents and their removal by evaporation will result in health hazards and environmental pollution. To address these problems, we employed a green chemical method to synthesize a new HNIW/TNT cocrystal. This method emphasizes that the organic solvent used is greener, safer and more environmentally benign.

Hence, in this current work we replace the toxic organic solvent (ethyl acetate) with a less-toxic solvent, ethanol, to reduce environmental destruction and damage to health. Because HNIW (also known as CL-20) is slightly soluble (0.503 g per 100 ml at 25°C) in ethanol (Holtz *et al.*, 1994 ▸), grinding was introduced to assist dissolution. Due to a faster and cleaner dynamic kinetic resolution, HNIW is dissolved in ethanol (at least to some extent) with the assistance of grinding. Moreover, compared with the ethyl acetate solvent (40.6 g CL-20 per 100 ml ethyl acetate at 25°C; Holtz *et al.*, 1994 ▸), the crystals are easier to grow from a poor solvent (ethanol) during the slow evaporation from solution (Xiao *et al.*, 2014 ▸). So, this green chemical approach using the less-toxic poor solvent ethanol is significantly different from the commonly used slow evaporation from solution and is also a better choice for growing the new HNIW/TNT cocrystal. In this paper the elemental change, crystal structure, explosive properties and the safety properties of the new cocrystal were investigated in detail.

## 2. Experimental   

### 2.1. Materials   

HNIW and TNT were provided by Modern Chemistry Research Institute of China. Ethanol was purchased from Tianjin Hengxing Chemical Co. Ltd of China.

### 2.2. Preparation of HNIW/TNT cocrystals by green chemical methods   

HNIW (4.38 g, 10 mmol) and TNT (2.27 g, 10 mmol) were introduced into a grinding jar with a large quantity (133 g) of zirconia grinding balls (diameter 0.2 mm), followed by 33.3 ml of ethanol. The jar was then quickly closed to prevent volatilization of ethanol. Grinding was performed for 2 h at 3000 rpm on a HWNSM-0.3L grinder (Shanghai HWEI Mechanical & Electrical Equipment Co., Ltd) at 220 V and 50 Hz. The experiment was performed at room temperature. Single crystals of the new HNIW/TNT cocrystal were obtained by slow evaporation of ethanol filtrate containing the material prepared by grinding.

In this experiment, ethanol was used as an organic solvent, which is less toxic and is a better choice for our original aim, green chemistry. Furthermore, a grinder was used to assist dissolution of HNIW. It highlights a faster and cleaner dynamic kinetic resolution and grind technology as a scalable, sustainable and environment friendly strategy. Application of the grind techniques can reduce handling time, decrease reaction temperature, circumvent toxic solvents and reactant, minimize production costs, improve reaction rates and increase reaction yield. Therefore, our experiment is conducive to reduce environment destruction and damage to health. It further executes the green behavior.

**Figure 1:**
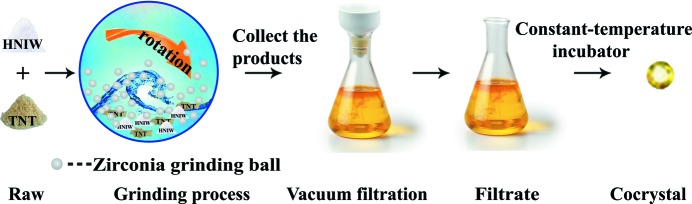
Schematic illustration of the set-up for the green chemical method synthesis of the new cocrystal.

A general synthesis schematic of the green chemical process is shown in Fig. 1 ▸.

### 2.3. Characterizations methods   

#### 2.3.1. Scanning electron microscopy   

The particle morphology, size distribution, crystal structure and composition analysis of the new HNIW/TNT cocrystals and the co-formers were examined using a MIRA3 LMH scanning electron microscope (Tescan, Czech Republic).

#### 2.3.2. Single-crystal X-ray diffraction (SXRD)   

SXRD data of the new HNIW/TNT cocrystals with suitable quality were collected using a Bruker Smart Apex-II X-ray single-crystal diffractometer. The instrument was equipped with graphite-monochromated Mo *K*α radiation (λ = 0.71073 Å) at 50 kV and 30 mA. A total of 21696 reflections was collected (−11 ≤ *h* ≤ 11, −19 ≤ *k* ≤ 22, −29 ≤ *l* ≤ 28), of which there were 4020 unique reflections (*R*_int_ = 0.0518). The crystal structure was refined by full-matrix least-squares methods, and absorption correction was semi-empirical from equivalents. All hydrogen atoms were refined anisotropically and fixed at 0.93 Å. Non-hydrogen atoms were refined using anisotropic thermal parameters.

#### 2.3.3. Powder X-ray diffraction (PXRD)   

PXRD data of the new HNIW/TNT cocrystals and the raw materials on lightly ground bulk materials were collected with a DX-2700 powder X-ray diffractometer (Dandong Haoyuan Corporation, Liaoning, China) operating at a tube voltage of 40 kV and a current of 30 mA using Cu *K*α radiation at λ = 1.5418 Å. The patterns were scanned in the range 5 ≤ θ ≤ 50° with a 0.03° step size. Additionally, PXRD patterns were analyzed using *Jade 9* (Materials Data Inc., Livermore, CA, USA) and *PDF-2* (International Centre for Diffraction Data, 2009 ▸) software.

#### 2.3.4. Fourier transform infrared (FT–IR) spectroscopy   

FT–IR was performed using a Spectrum 100 infrared spectrometer (Perkin-Elmer, Inc.). Samples were prepared as KBr disks and spectra were recorded over the range 400–4000 cm^−1^ with a resolution of 4 cm^−1^. Each spectrum was the average of 32 individual scans.

#### 2.3.5. Raman spectroscopy   

The Raman spectra of the new HNIW/TNT cocrystals and the raw materials were recorded using a LabRAM HR800 laser confocal micro-Raman spectrometer (Horiba Jobin Yvon Corporation, France).

#### 2.3.6. X-ray photoelectron spectroscopy   

The element of the samples was traced by a Thermo ESCALAB 250XI X-ray photoelectron spectrometer (Thermo Fisher Scientific, America).

#### 2.3.7. Differential scanning calorimetry   

Thermal behavior of the samples was measured using a differential scanning calorimeter (Setaram DSC-131, Setaram Corporation, France) by heating 0.7 mg of HNIW/TNT cocrystals in a hermetically sealed aluminium crucible with a pinhole in the lid at a heating rate of 10°C min^−1^. The variable-temperature DSC curves for HNIW, new HNIW/TNT cocrystals and TNT were recorded from 25 to 325°C under nitro­gen flow (30 ml min^−1^).

#### 2.3.8. Detonation velocity   

**Figure 2:**
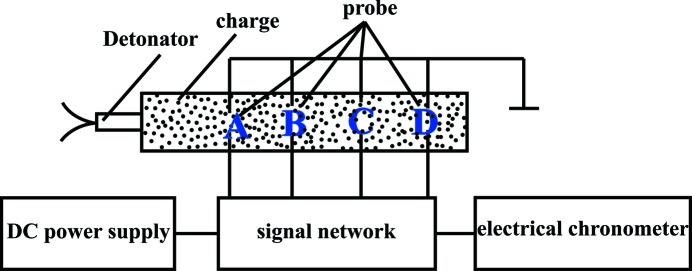
Schematic of detonation velocity measurement using a timing method.

Detonation velocity is an important parameter to evaluate the energetic performance of the HNIW/TNT cocrystals, which was tested by a timing method. A schematic of the detonation velocity measurement is given in Fig. 2 ▸. A full description of the measurement technique is given in the supporting information.

#### 2.3.9. Mechanical sensitivity test   

**Figure 3:**
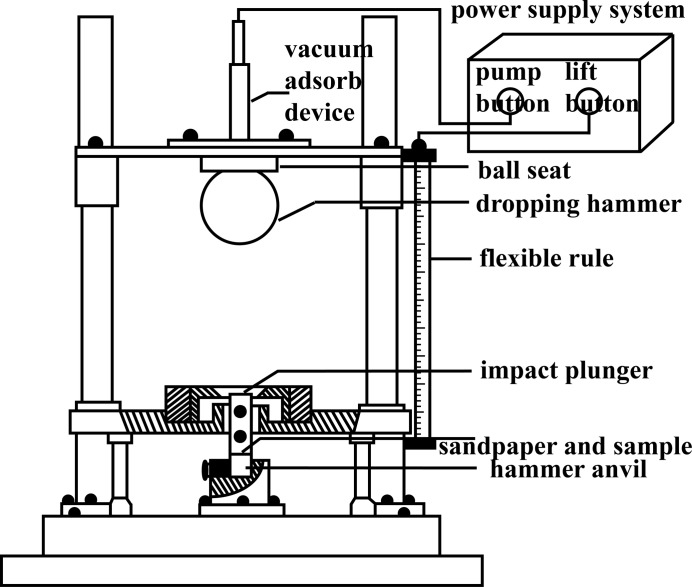
Experimental set-up for measuring impact sensitivity.

The impact sensitivity was determined with an ERL Type 12 drop-hammer apparatus according to GJB-772A-97 standard method 601.2 (National Military Standard of China, 1997 ▸). The experimental set-up for measuring impact sensitivity is shown in Fig. 3 ▸. The testing conditions are: drop weight of 2.500 ± 0.002 kg; sample mass of 35 ± 1 mg; ambient temperature of 20°C and relative humidity of 30%. The samples was placed on the hammer anvil and hit by a drop hammer. When the drop hammer dropped, the special height (*H*_50_) represented a critical drop height with 50% explosion probability. The test was carried out 30 times to characterize the impact sensitivity of the samples. A full description of the impact sensitivity experiment is given in the supporting information.

The friction sensitivity was tested on a WM-type friction sensitivity apparatus according to GJB-772 A-97 standard method 602.1 (National Military Standard of China, 1997 ▸). The testing conditions are as follows: pendulum weight of 1.50 ± 0.01 kg, sample mass of 20 ± 1 mg, relative pressure of 4.9 MPa, swaying angle of 90°, ambient temperature of 20°C and relative humidity of 30%. The friction sensitivity of each test sample was expressed by explosion probability (*P*). The test was carried out continuously 30 times to characterize the friction sensitivity and explosion probability of the samples.

**Figure 4:**
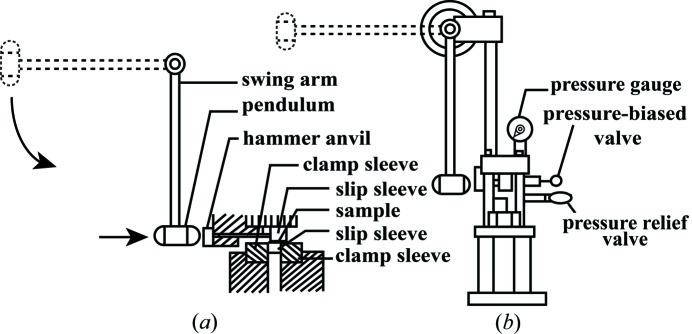
Experimental set-up for measuring friction sensitivity.

The experimental set-up for measuring the friction sensitivity is shown in Fig. 4 ▸. A full description of the friction sensitivity experiment is given in the supporting information.

## 3. Results and discussion   

### 3.1. Scanning electron microscopy (SEM)   

**Figure 5:**
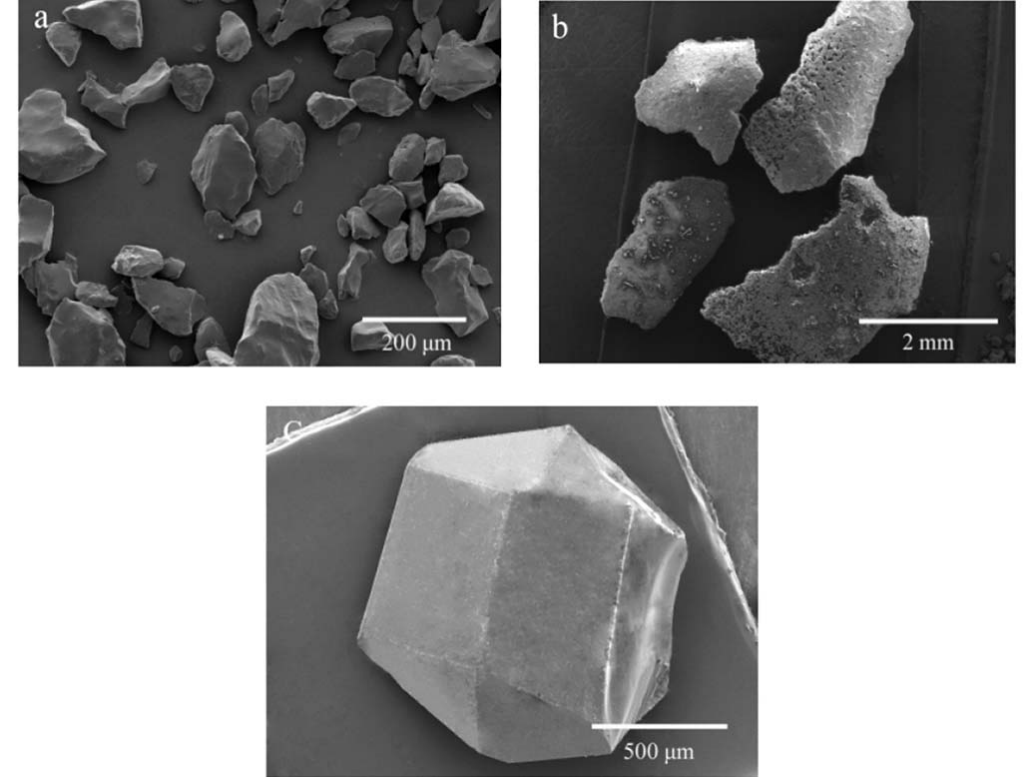
SEM micrographs of crystals of (*a*) raw HNIW, (*b*) TNT and (*c*) the new HNIW/TNT cocrystal.

Crystal quality (crystal shape, crystal size, crystal surface and crystal defects) plays an important role during storage, transport and use of explosives. These physicochemical parameters may affect the detonation initiation spots of the explosive (Shen *et al.*, 2011 ▸). The morphological differences of HNIW, TNT and the synthesized HNIW/TNT cocrystal are illustrated in Fig. 5 ▸.

HNIW of about average particle size 100 µm possesses a hippocrepiform-type crystal morphology with sharp crystal edges; nevertheless, many crystal defects such as dislocations and grain boundaries were observed to exist on the crystal round (Fig. 5 ▸ *a*). TNT is a irregular flake (Fig. 5 ▸ *b*) with a coarse surface and lots of particle deposits appear on the surface. The dimensions of the flake range from 2 to 3 mm. In contrast, the surfaces of the new HNIW/TNT cocrystals are smooth, their shapes are regular and their form is hexahedral, as shown in Fig. 5 ▸(*c*). The particle size of the HNIW/TNT cocrystals is about 1 mm, which is clearly large than for HNIW. Their different morphologies are most likely to affect their physicochemical properties (Yang *et al.*, 2014 ▸). The above results unambiguously indicate that the new HNIW/TNT cocrystal possesses a unique crystal shape and crystal size that is unlike HNIW and TNT. The cocrystal strategy not only drastically alters the crystal shape but also the crystal size.

### 3.2. Single-crystal X-ray diffraction (SXRD)   

**Figure 6:**
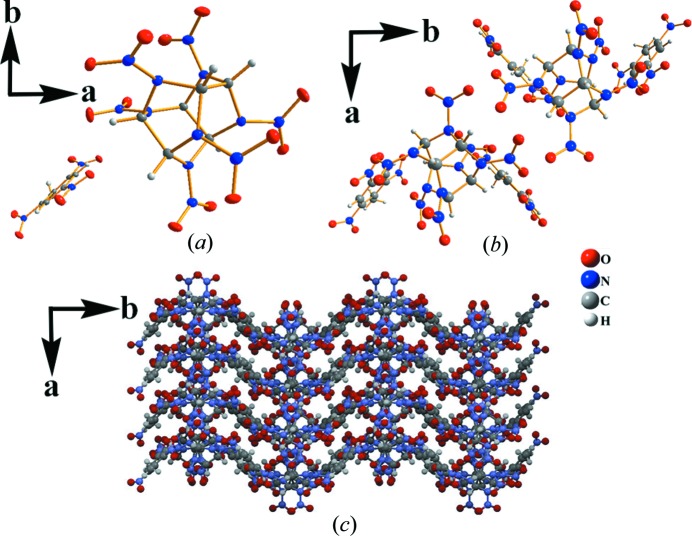
The new HNIW/TNT cocrystal: (*a*) *ORTEP* view, (*b*) view down the *c* axis of the one-dimensional layered structure and (*c*) view down the *c* axis of the three-dimensional arrangement.

The structure of the new HNIW/TNT cocrystals was determined by SXRD. The packing arrangement is presented in Fig. 6 ▸. Detailed crystallographic data and structure refinement parameters for the new HNIW/TNT cocrystals and the previous reported HNIW/TNT cocrystal (Yang *et al.*, 2013 ▸) are summarized in Table 1 ▸. The hydrogen bond geometry between the HNIW and TNT in the new cocrystal structure and the previously reported cocrystal (Yang *et al.*, 2013 ▸) are given in Table 2 ▸.

The new cocrystal structure comprises two independent molecules (HNIW and TNT) and it is an asymmetric unit Fig. 6 ▸(*a*). In the new cocrystal, three cooperative hydrogen bonds link adjacent HNIW and TNT molecules, forming one-dimensional chains along the *c*-axis direction [Fig. 6 ▸(*b*) and Table 2 ▸). The one-dimensional chained structure extends infinitely in two directions, forming a two-dimensional network. Then, the two-dimensional networked structure stacks continuously, generating a three-dimensional arrangement along the *c*-axis direction (Fig. 6 ▸ *c*). The three-dimensional structure is a sine wave pattern and propagates in the crystal.

Table 2 ▸ shows that three hydrogen bonds exist between molecules of HNIW and TNT by which the new HNIW/TNT cocrystals assemble into a two-component cocrystal. However, only two hydrogen bonds (C—H8⋯O3 and C—H5⋯O13) exist in the previous cocrystal. This interaction of hydrogen bonds is assumed to be reason for the formation of the new cocrystals. It is commonly noted in other energetic cocrystals and may be an important consideration in the design of future materials (Bolton *et al.*, 2012 ▸). Other differences from the previous cocrystal are hydrogen bond lengths and bond angles. A different crystal structure shows different macroscopic morphology and different properties. Thus, the color, shape and crystal density are different, which can be seen from Table 1 ▸.

In the new structure, the crystal is light yellow and a hexahedron. However, in the structure reported previously, the crystal is colorless and a prism. Aside from color and shape, the density is also significantly improved to that published previously. In the new structure, the density is 1.934 g cm^−3^, which is an increase of ∼5% on the previously reported density (1.864 g cm^−3^). This result further illustrates that different crystal structure does make macroscopic morphologies and properties.

Particularly, in the field of energetic materials, HNIW (CL-20) possesses a maximum crystal density of 2.035 g cm^−3^ (only for ∊-CL-20) and aside from a few instances, the others are less than 2 g cm^−3^ (Zhang *et al.*, 2017 ▸). So, making the density value of energetic materials more than 2 g cm^−3^ is a huge challenge due to its inherent nature. Analogously, a great challenge also appears at more than 1.9 g cm^−3^. Therefore, 1.934 g cm^−3^ in the new HNIW/TNT cocrystal is a great breakthrough in energetic materials.

The new crystal structure also shows other different properties such as DSC, detonation velocity and mechanical sensitivity. This has been proven by more stable thermal behavior, higher detonation velocity and safer mechanical sensitivity. Aside from these three properties reported by Yang *et al.* (2013 ▸), we report additional studies (PXRD, FT–IR, Raman spectroscopy and XPS).

### 3.3. Powder X-ray diffraction (PXRD)   

**Figure 7:**
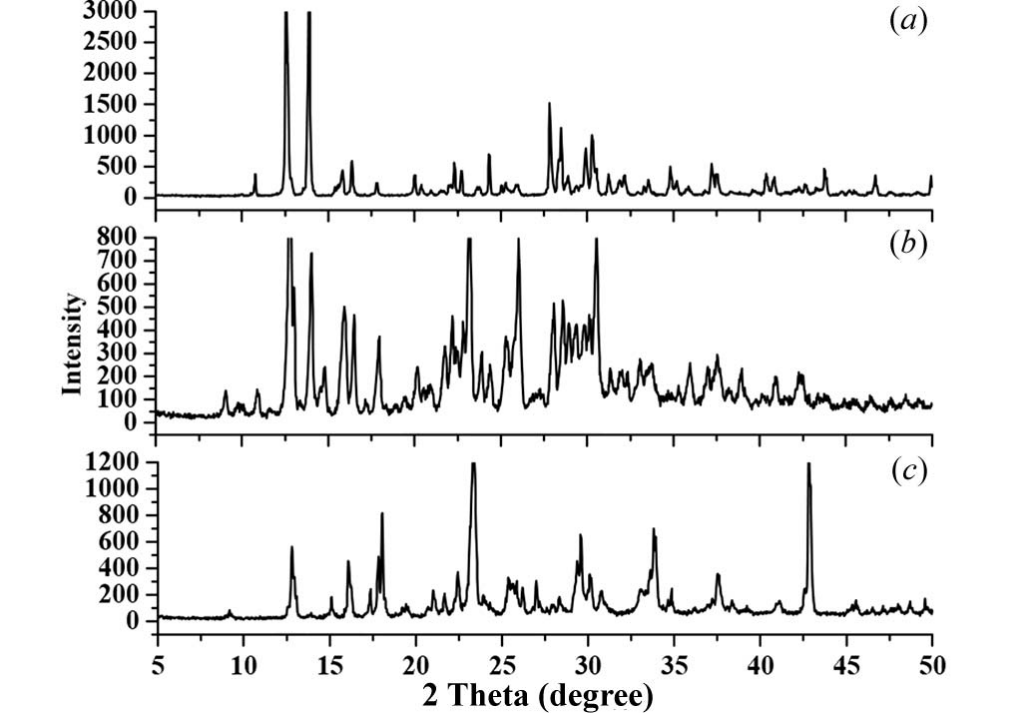
PXRD diffractogram of (*a*) HNIW, (*b*) new HNIW/TNT cocrystals and (*c*) TNT.

X-ray diffraction (PXRD) measurements were carried out to identify whether a new polymorphism in new HNIW/TNT cocrystals (Xiong *et al.*, 2017 ▸). PXRD analysis of the HNIW, new cocrystals, and TNT gave a diffractogram in Fig. 7 ▸.

The pattern reveals that the new HNIW/TNT cocrystal exhibits unique peaks at 2θ ≃ 15.97°, 26.04°, 28.09° and 30.57°, which is evidently different from those of the co-formers. Simultaneously, in the 2θ (°) ranges 21.69–23.14, 25.27–26.05 and 28.09–30.56, the diffraction intensity of the new HNIW/TNT cocrystals is much stronger than the coformers. It can also be observed that the main sharp peaks for the HNIW at 2θ ≃ 27.84°, and for the TNT at 2θ ≃ 33.93° and 42.83° disappear in the pattern of the new cocrystal. The weak or disappeared diffraction intensity of the original diffraction peaks and the strong or occurred diffraction intensity of the new diffraction peaks clearly demonstrate that the synthesized material by a green chemical method completely converts to the new HNIW/TNT cocrystals form and is not a product of crystal transformation or a simply physical accumulation by HNIW and TNT particles at a macroscopic scale, but they have interacted to change inherent structure and component forming a new crystal. Good agreement with the results of single crystal showed that a new HNIW/TNT cocrystals structure was obtained by green chemically synthesized.

### 3.4. Fourier transform infrared (FT–IR) spectroscopy   

**Figure 8:**
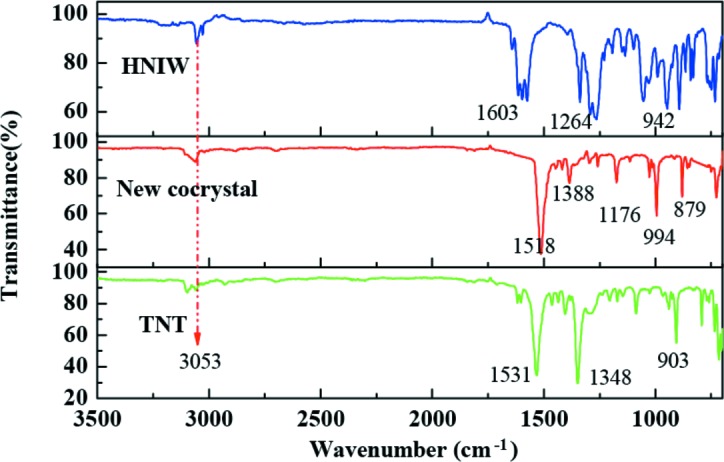
IR spectra of the samples.

FT–IR analysis was employed to identify the molecular structure of the HNIW, new HNIW/TNT cocrystals and TNT, and the results are displayed in Fig. 8 ▸. In the IR spectrum of the new HNIW/TNT cocrystals, the absorption peaks located at 3053 cm^−1^ and 1518 cm^−1^ correspond to the stretching vibrations of C—H and N—NO_2_, respectively. This means that in the cocrystallization process, the –CH (in TNT) reacted with N—NO_2_ (in HNIW) to form a hydrogen bond, and this observation agrees with the SXRD data. The peak at 1176 cm^−1^ reflects the stretching vibrations of C—N. Peaks at 994 cm^−1^ and 879 cm^−1^ reflect the deformation vibrations of ring vibrations of TNT and the –NO_2_ (in HNIW), respectively. Overall, the change of the IR absorption peaks in the new cocrystal is due to the formation of the hydrogen bond. The results indicate that the introduction of a donor–acceptor interaction between HNIW and TNT represents a reliable supramolecular synthon for the formation of new HNIW/TNT cocrystals, which has a profound influence on the packing of the molecules in the cocrystals of the energetic material (Landenberger & Matzger, 2010 ▸).

### 3.5. Raman spectroscopy   

**Figure 9:**
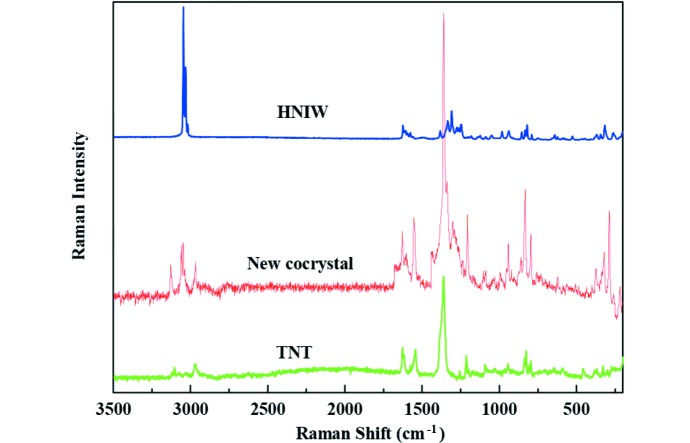
Raman spectra of HNIW, new HNIW/TNT cocrystal and TNT.

To explore the reason of cocrystal formation, Raman analysis was carried out. The Raman spectra and the assignment of the major vibrational bands for the HNIW, new HNIW/TNT cocrystal and TNT in the region 200–3500 cm^−1^ are shown in Fig. 9 ▸ and Table 3 ▸, respectively. The results indicated that in the new HNIW/TNT cocrystal, a large number of characteristic peaks shifted markedly. From Fig. 9 ▸, we can see that compared with HNIW and TNT, peaks in the new cocrystal are obvious and sharp, mainly over the region 750–1700 cm^−1^. In particular, the C—H stretching vibration in HNIW at 3049.6, 3033.0 and 3024.7 cm^−1^ shifted to 3127.8, 3044.1 and 3037.2 cm^−1^ in the new HNIW/TNT cocrystal. The NO_2_ asymmetric stretching in TNT at 1628.6 cm^−1^ shifted to 1623.8 cm^−1^ in the new HNIW/TNT cocrystal. The shift of these characteristic vibrational bands are due to hydrogen bond C13—H13*A*⋯O1 between HNIW and TNT. Furthermore, the C—H stretching vibration in TNT at 1360.9 cm^−1^ shifted to 1362.4, 1347.1 and 1340.1 cm^−1^ in new HNIW/TNT cocrystal. The NO_2_ asymmetric stretching in HNIW at 1630.7, 1623.8, 1609.9 and 1596.1 cm^−1^ shifted to 1672.2 cm^−1^ in the new HNIW/TNT cocrystal. This phenomenon can be attributed to hydrogen bonds C2—H2*A*⋯O10 and C4—H4*A*⋯O14 between HNIW and TNT. These results are consistent with the SXRD analysis.

### 3.6. X-ray photoelectron spectroscopy (XPS)   

**Figure 10:**
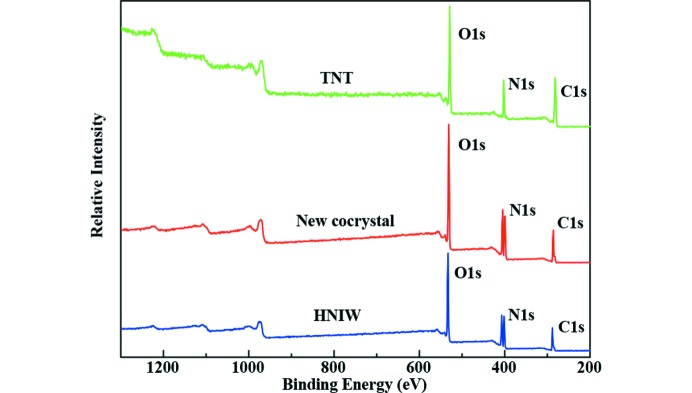
XPS survey spectra of HNIW, new HNIW/TNT cocrystal and TNT.

To trace the elemental change of HNIW and TNT and the new cocrystal, XPS analysis was employed, and the XPS spectra are shown in Figs. 10 ▸, 11 ▸, 12 ▸ and 13 ▸.

**Figure 11:**
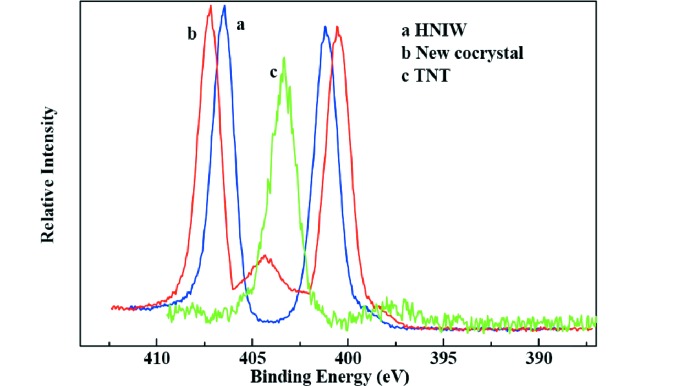
High-resolution detail of N 1*s* peak of HNIW, new HNIW/TNT cocrystal and TNT by XPS.

**Figure 13:**
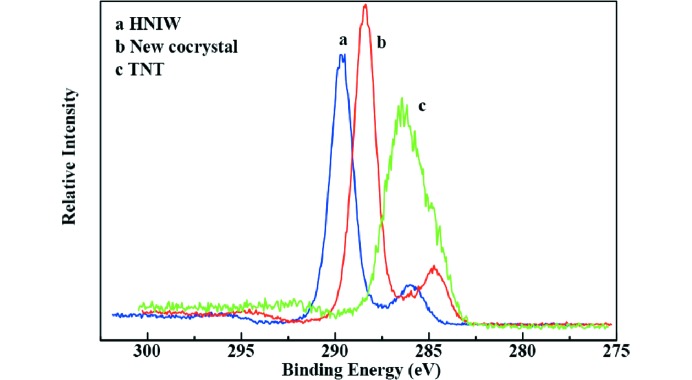
High-resolution detail of C 1*s* peak of HNIW, new HNIW/TNT cocrystal and TNT by XPS.

The survery spectra illustrated in Fig. 10 ▸ show that three elements (N 1*s*, O 1*s* and C 1*s*) were detected in each sample. By analysis of the N 1*s* spectra of three samples in Fig. 11 ▸, two peaks appeared in HNIW with binding energy of 401.16 and 406.47 eV, respectively, which arises from C—N—C and N—NO_2_, respectively. Only one peak occurred in TNT with binding energy of 403.41 eV, attributing to C—NO_2_. However, there are three peaks in the new HNIW/TNT cocrystal with binding energies of 400.56, 404.43 and 407.29 eV. It can be seen that significant shift was observed from 401.16 to 400.56 eV and 406.47 to 407.29 eV of the two peaks in HNIW. Meanwhile, a shift from 403.41 to 404.43 eV of one peak was observed in TNT. High-resolution XPS shows one peak of O 1*s* centered at 533.49 eV in new HNIW/TNT cocrystal. This binding energy was a obvious shift, comparing with 534.32 eV in HNIW and 532.04 eV in TNT. Due to an analogous chemical environment for —NO_2_, the shapes of all the O 1*s* peaks are similar. Fig. 13 ▸ shows the C 1*s* spectra of HNIW, new cocrystal and TNT, in which the characteristic peaks are at the binding energies of 289.69, 288.39 and 286.46 eV, respectively. The reasons for binding energy are due to C—N in HNIW and C—C or C—NO_2_ in TNT. However, the peak shifts markedly in the new HNIW/TNT cocrystal. In the three high-resolution XPS spectra we can clearly see that due to the hydrogen bonds, the position and bind energy of peaks in new cocrystal are obvious different from HNIW and TNT. Furthermore, no additional signal was detected in the spectrum, implying that no other functional groups are attached and the samples are very pure after green chemical synthesis.

### 3.7. Differential scanning calorimetry   

**Figure 14:**
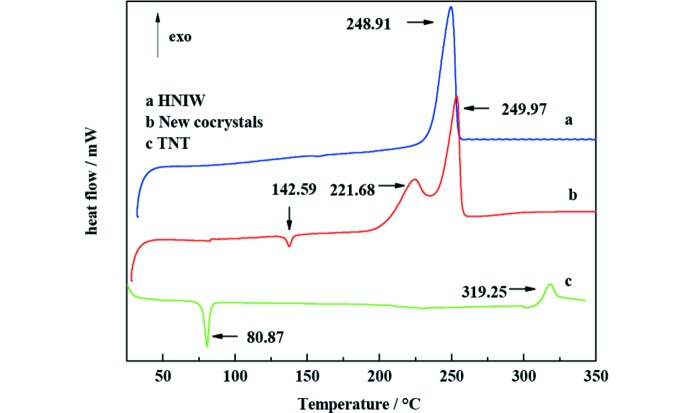
DSC thermograms of HNIW, new HNIW/TNT cocrystals and TNT heating rate of 10°C min^−1^.

The thermal behavior of HNIW, new HNIW/TNT cocrystals and TNT was examined by differential scanning calorimetry (DSC). DSC thermograms of HNIW, new HNIW/TNT cocrystals and TNT are shown in Fig. 14 ▸ and the experimental results are presented in Table 4 ▸.

HNIW gives a strong exothermic peak at 248.91°C attributed to the decomposing of the crystal (Yang *et al.*, 2012 ▸; Foltz *et al.*, 1994 ▸). TNT has an endothermic peak at 80.87°C, corresponding to its melting point (Shamim *et al.*, 2015 ▸). Furthermore, a unique exothermic peak is observed at 319.25°C. However, the endothermic peak of the new HNIW/TNT cocrystals locates at 142.59°C; simultaneously, there are two obvious exothermic decomposition peaks. The first presents at 221.68°C and the second appear at 249.97°C, which correspond to low-temperature decomposition and high-temperature decomposition, respectively (Cheng *et al.*, 2016 ▸). Consequently, it is notable that some significant changes are given in the thermal properties of new HNIW/TNT cocrystals and a distinct thermal behavior is presented in the endothermic decomposition and exothermic decomposition. Furthermore, the stability of the new cocrystal is improved due to the hydrogen bonding in the structure. These results demonstrate that such a new cocrystal structure *via* a green chemical process provides an efficient route for excellent thermal property and stability.

### 3.8. Detonation velocity   

To evaluate explosive performance, the detonation velocities of HNIW, new HNIW/TNT cocrystals, the previously reported cocrystal and TNT were measured using a timing method. The detonation velocity (*D*) is calculated as *L*/*t*, where *L* is the height of the explosive cylinders and *t* is the travel time of the detonation wave between two probes. Because the values of *t* and *L* exhibit some difference during the six successful tests, the final apparent detonation velocity 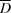 of each sample is expressed as the average value, in this case from six successful tests.

**Figure 15:**
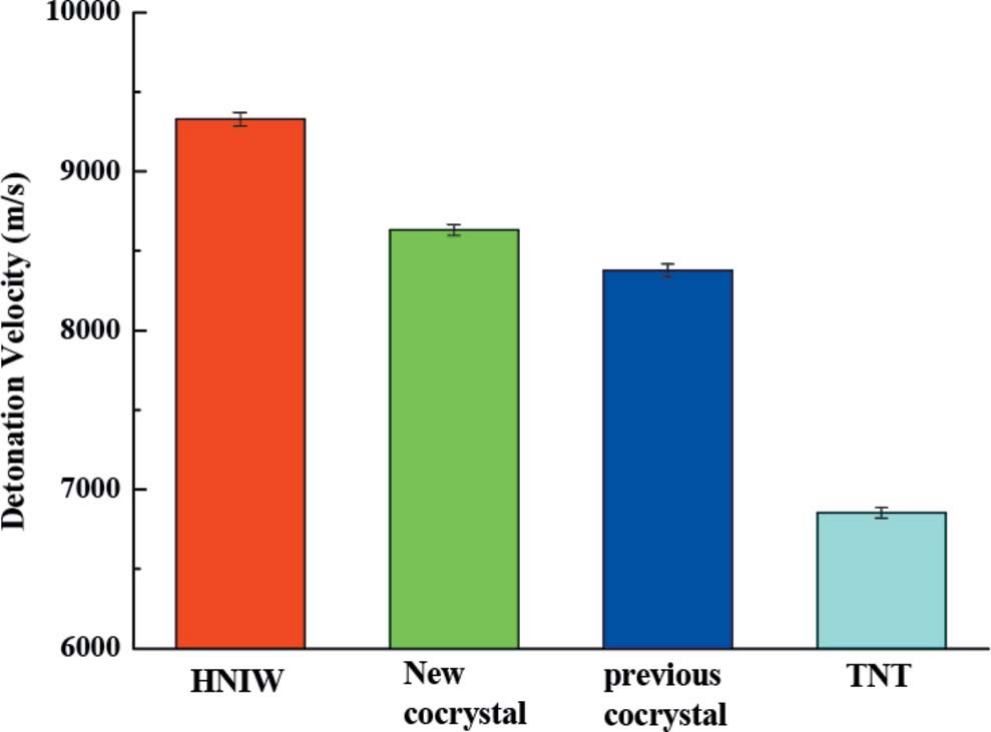
The 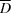 values of HNIW, cocrystals and TNT as measured by a timing method. The error bars represent the standard deviation for the average value obtained from six effective tests.

The 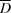 values for HNIW, new HNIW/TNT cocrystal, previously reported cocrystal and TNT are presented in Fig. 15 ▸. The detonation velocity of the new cocrystal (8631 m s^−1^) in our manuscript is higher than for the previously reported cocrystal (8426 m s^−1^; Yang *et al.*, 2013 ▸) and this result is also higher than for TNT (6854 m s^−1^). The reason can be explained as follows. In the field of energetic materials, the density value seriously affects detonation velocity. Increased crystallographic density can decrease the volume and the number of cavities in the crystal structure. Therefore, when the volume of explosive cylinders remains constant, high density leads to high mass. Under the same conditions, the higher the mass of the explosive, the greater the energy ouput and the higher the detonation velocity, *i.e.* under the same conditions, a higher density energetic material shows a promising detonation velocity (Zhang *et al.*, 2017 ▸). Due to the higher density (1.934 g cm^−3^ *versus* 1.846 g cm^−3^, see Table 1 ▸), a higher detonation velocity has been measured in the new cocrystal.

Although the HNIW, with a detonation velocity of 9329 m s^−1^, is more powerful than new HNIW/TNT cocrystals, it is obvious that HNIW possesses high sensitivity and that occurs for phase transition which is accompanied by the increase in temperature. For example, all polymorphs of HNIW convert to the γ-form once heated above 150°C (Foltz *et al.*, 1994 ▸). In contrast, new HNIW/TNT cocrystals are more thermodynamically stable than HNIW, which is in accordance with data in Fig. 14 ▸.

The results show that the introduction of the green chemical methods to cocrystallize HNIW with TNT can improve its detonation property by the interaction of hydrogen bonds. This is helpful to increase the detonation properties of energetic materials.

### 3.9. Mechanical sensitivity test   

The safety performance of HNIW, new HNIW/TNT cocrystals, and TNT is investigated by estimation the impact and friction sensitivities for stimuli. The results are listed in Table 5 ▸.

The special height (*H*_50_) and explosion probability (*P*) of the new cocrystals are 43 ± 0.02 cm and 49%, respectively, which indicates that the *H*_50_ value increases by 231% and *P* has an obvious decrease of 51% for the new HNIW/TNT cocrystal reflecting a evidently lower mechanical sensitivity than HNIW. The reason is that the HNIW and TNT molecules are packed compactly and uniformly through intermolecular interactions of hydrogen bonds and other directional interactions during cocrystallization (Wei *et al.*, 2015 ▸). This packing stabilizes the structure and efficiently reduces the volume and number of voids in the crystal structure, therefore leading to a reduction in the probability of formation hot spots under impact and friction stimuli. The explosion of an explosive is due to the formation of hot spots under extrinsic stimulation (Field, 1992 ▸; Wang *et al.*, 2016 ▸). So, the new stable cocrystal structure can dramatically improve the mechanical sensitivity.

The results illustrate that the new cocrystal is more insensitive to mechanical stimuli due to its unique crystal structure formed by cocrystallization. When the new cocrystal undergoes an external stimulation action, the close and uniform molecule structure acts as a buffer system to dissipate the impact and friction energy. It demonstrates that an obvious desensitization effect has been achieved for the prepared new HNIW/TNT cocrystals *via* a green chemical method.

## 4. Conclusion   

Herein, we report a new HNIW/TNT cocrystal with higher density and better properties than previous reported (Yang *et al.*, 2013 ▸) for the cocrystal, which was prepared *via* a green chemical method. The results indicate microscopically and macroscopically that the cocrystal is a new structure due to the new set of hydrogen bonds between the HNIW and TNT. Our investigations showed that this new cocrystal structure possesses preferable crystal quality, more stable thermal property and lower mechanical sensitivity. In particular, the crystal density and detonation velocity are excellent compared with those of the cocrystal reported by Yang *et al.* (2013) ▸. This synthesis is environmentally benign, scalable and a sustainable strategy.

Moreover, these results also make it clear that with the assistance of grinding, a poor solvent can be employed in a solvent evaporation process. This technological innovation indicates the transformative potential of the solvent evaporation method in the crystal-growing lexicon and should stimulate more research to further its systematic development. This study should also encourage further studies into the design and synthesis of more functionally interesting cocrystal materials.

## Supplementary Material

Crystal structure: contains datablock(s) I. DOI: 10.1107/S2052520618008442/lo5028sup1.cif

Structure factors: contains datablock(s) I. DOI: 10.1107/S2052520618008442/lo5028Isup2.hkl

Supporting information file. DOI: 10.1107/S2052520618008442/lo5028sup3.pdf

CCDC reference: 1823458

## Figures and Tables

**Figure 12:**
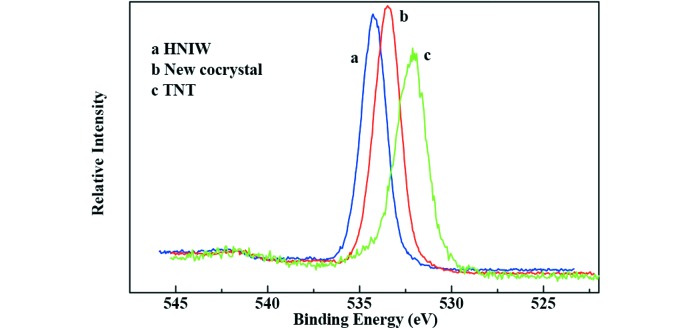
High-resolution detail of O 1*s* peak of HNIW, new HNIW/TNT cocrystal, and TNT by XPS.

**Table 1:** Crystal data and structure refinement parameters

	New cocrystal structure	Cocrystal reported by Yang *et al.* (2013 ▸)
Chemical formula	C_13_H_11_N_15_O_18_	C_13_H_11_N_15_O_18_
Formula weight	665.37	665.37
Color	Light yellow	Colorless
Morphology	Hexahedron	Prism
Temperature (K)	296	293.15
Crystal system	Orthorhombic	Orthorhombic
Space group	*Pbca*	*Pbca*
*a* (Å)	9.6268 (12)	9.7352 (2)
*b* (Å)	19.292 (2)	19.9121 (6)
*c* (Å)	24.606 (3)	24.6955 (6)
Unit-cell volume (Å^3^)	4569.8 (10)	4787.20 (10)
*Z*	8	8
Calculated density (g cm^−3^)	1.934	1.846
Absorption coefficient (mm^−1^)	0.181	-
*F*(000)	2704	-
θ_min_, θ_max_ (°)	2.11, 25.00	6.08, 52.74
No. of reflections collected	21696	12659
No. of independent reflections	4020	4892
No. of data, restraints, parameters	4020, 0, 416	-
Goodness-of-fit	1.083	1.023
Final *R*1, *wR*2 indices [*I* > 2σ(*I*)]	0.0372, 0.0990	0.0489, 0.1166
*R*1, *wR*2 indices (all data)	0.0429, 0.1022	-
Extinction coefficient	0.0011 (2)	-
CCDC No.	1823458	-

**Table 2:** Hydrogen bond lengths and bond angles between HNIW and TNT in the new structure and the previously reported structure

C—H⋯O	H⋯O (Å)	C⋯O (Å)	C—H⋯O (°)
New structure			
C2—H2*A*⋯O10^i^	2.51	3.427 (2)	168
C4—H4*A*⋯O14^ii^	2.44	3.316 (2)	157
C13—H13*A*⋯O1^iii^	2.37	3.173 (2)	138
Previously reported structure†			
C—H8⋯O3	2.31	–	146
C—H5⋯O13	2.62	–	161

Symmetry codes: (i) 1 − *x*, −*y*, 1 − *z*; (ii) *x*, 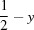, 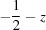; (iii) 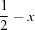, −y, 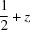.

† Yang *et al.* (2013 ▸).

**Table 3:** Assignments of the major bonds from 200 to 3500 cm^−1^ of the Raman spectra of samples

Assignment	HNIW (cm^−1^)	New HNIW/TNT (cm^−1^)	TNT (cm^−1^)
C—H stretching	3049.6	3127.8	3107.0
	3033.0	3044.1	
	3024.7	3037.2	
CH_3_ stretching		2965.1	2967.9
N—N stretching (N—NO_2_)		1672.2	
NO_2_ asymmetric stretching	1630.7		
	1623.8		
	1609.9		
	1596.1		
	1575.3		
C—N stretching (C—NO_2_)		1623.8	1628.6
C≡C stretching		1554.6	1547.7
	1540.8		
C—H stretching	1381.6	1362.4	1360.9
	1305.5	1347.1	
	1340.1		
C—C stretching	1249.5	1201.1	1214.9
	1235.7		1104.2
	1221.8		
C—H deformation		1097.3	
	1035.0		
	993.5		
Ring stretching		965.8	945.1
	938.2		
	924.3		
Ring deformation		862.1	840.6
		819.9	
C—C stretching	826.8	822.5	
	806.0	799.1	
	785.3		
	757.7		
Cage deformation	293.4	369.5	
	231.1	314.2	
	286.5		
	217.3		

**Table 4:** Summarized data from DSC thermograms of HNIW, new HNIW/TNT cocrystals and TNT

Specimen	Melting point (°C)	Endothermic decomposition (°C)	Exothermic decomposition (°C)
HNIW	-	-	248.91
New HNIW/TNT	-	142.59	221.68, 249.97
TNT	80.87	-	319.25

**Table 5:** Results of the mechanical sensitivity test for HNIW, new HNIW/TNT cocrystals and TNT

Samples	Impact sensitivity		Friction sensitivity	
	*H*_50_ (cm)	Drop (%)	*P* (%)	Drop (%)
Raw HNIW	13	-	100	-
New cocrystals	43	↑231	49	↓51
Raw TNT	95	-	38	-
