# Supplementary material for: High-density HNIW/TNT cocrystal synthesized using a green chemical method
Source: Acta Crystallogr B Struct Sci Cryst Eng Mater. 2018 Jul 23;74(Pt 4):385–93. doi: 10.1107/S2052520618008442 (PMC6108158; doi:10.1107/S2052520618008442)
Supplement: Supplementary file 3 [file b-74-00385-sup3.pdf]

Acta Cryst B

STRUCTURAL SCIENCE
CRYSTAL ENGINEERING
MATERIALS

Volume 74 (2018)

Supporting information for article:

High-density HNIW/TNT cocrystal synthesized using a green chemical method

Yan Liu, Chongwei An, Jin Luo and Jingyu Wang

S1. Detonation velocity measurement

The experimental procedure of detonation velocity was as follows: the HNIW/TNT cocrystal explosive was molded as explosive cylinders. The suitable explosive cylinders were placed one by one in the sleeve. The sleeve filled with the explosive cylinders was jointed with a detonator and probe and placed in an explosion box. The assembled sample was connected with a DC power supply, signal network and an electrical chronometer. The DC power supply was turned on to detonate the detonator. The explosive cylinder was initiated by the detonator. The travel time of the detonation wave (t) between two probes was recorded by an electrical chronometer. The distance of the two probes (L) is the height of the explosive cylinder, which was measured. Finally, the detonation velocity was calculated from t and L. The test was carried out 10 times and the number of successful tests was not less than 6.

S2. Mechanical sensitivity test

S2.1. Impact sensitivity

The procedure was as follows: the weighed sample flowed out through a cone funnel at a constant speed and piled up on the center of the sandpaper to form a pyramid. The sandpaper with the sample was then carefully placed at the center of the anvil and the impact plunger was gently pressed against the sample. The power supply was turned on, the lift button pressed and the desired height of the ball seat adjusted (the height can be obtained from the flexible rule). The pump button was then turned on and the ball seat was pumped into a vacuum. The dropping hammer was adsorbed at the desired height, when it was near the ball seat. The pump button was turned off. The dropping hammer then became free and started to free fall until it hit the impact plunger. Then the sample on the sandpaper was impacted to explode by the impact plunger. The hammer anvil was used to buffer the residual impact after test. When the dropping hammer fell from the setting height, the sample exploded. This setting height is the special height H_50.

S2.2. Friction sensitivity

The procedure was as follows: the weighed sample was carefully placed between two slip sleeves and the clamp sleeve tightened. The pressure-biased valve was turned on and the pressured adjusted to 4.9 MPa. The hammer anvil was inserted and checked that it touched the clamp sleeve. The pendulum was lifted and then released at a swaying angle of 90deg. Finally, the hammer anvil was taken out and the pressure relief valve was turned on. The friction sensitivity test was completed. Then the explosion probability of the sample was obtained by calculating the proportion of the total number of explosions to the total number of experiments.
